# Castleman Disease of the Parotid Gland: A Report of a Case

**DOI:** 10.1155/2015/265187

**Published:** 2015-12-27

**Authors:** Fawaz Abo-Alhassan, Fatemah Faras, Jassem Bastaki, Mutlaq K. Al-Sihan

**Affiliations:** ^1^Department of Surgery, Al-Adan Hospital, Ministry of Health, 40188 Kuwait City, Kuwait; ^2^Department of ENT, Zain and Al-Sabah Hospitals, Ministry of Health, 40188 Kuwait City, Kuwait; ^3^Department of Pathology, Sabah Hospital and Kuwait Cancer Control Center, Ministry of Health, 40153 Kuwait City, Kuwait

## Abstract

Castleman disease is an extremely rare benign lymphoproliferative disorder of unknown etiology. It affects the lymphatic chain in anybody region, although the commonest site is the mediastinum. The head and neck region is the second most common site; however, the salivary glands are rarely affected. We report a case of a 29-year-old Asian lady who presented with a 2-year history of an enlarging left parotid mass. Histopathology of the excisional biopsy confirmed the diagnosis of Castleman disease.

## 1. Introduction

Castleman disease (CD) is a rare, benign lymphoproliferative disorder, first described in 1954 [1]. CD, generally, has no sex predilection and most commonly affects young adults between 15 and 35 years of age [2]. CD has been given different names, including giant lymph node hyperplasia, angiomatous lymph node hamartoma, angiofollicular lymph node hyperplasia, follicular lymphoreticuloma, and benign giant lymphoma. The different terminologies reflect the unknown cause of this disease. The disease can affect any lymph node in the body; however, the mediastinum is the most common region, accounting for 60% of cases. The head and neck region is involved in 14% [3], and between those 85% are occurring in the neck. Salivary gland involvement is extremely rare. From our literature review, we concluded that fewer than 30 cases of Castleman disease involving the parotid gland have been reported up-to-date. In this paper, we present a rare case of unicentric CD in the left parotid gland. Our patient was treated with surgical excision of the lesion and was followed up postoperatively.

## 2. Case Presentation

A 29-year-old Filipino female presented to our ENT clinic complaining of a swelling of two-year duration in the left parotid region. The swelling was progressively increasing in size for the past 6 months with no history of trauma and without any suspiciously related lesions elsewhere. On physical examination, there were no signs of inflammation, no palpable lymph nodes, and no evidence of facial nerve involvement.

**Figure 1:**
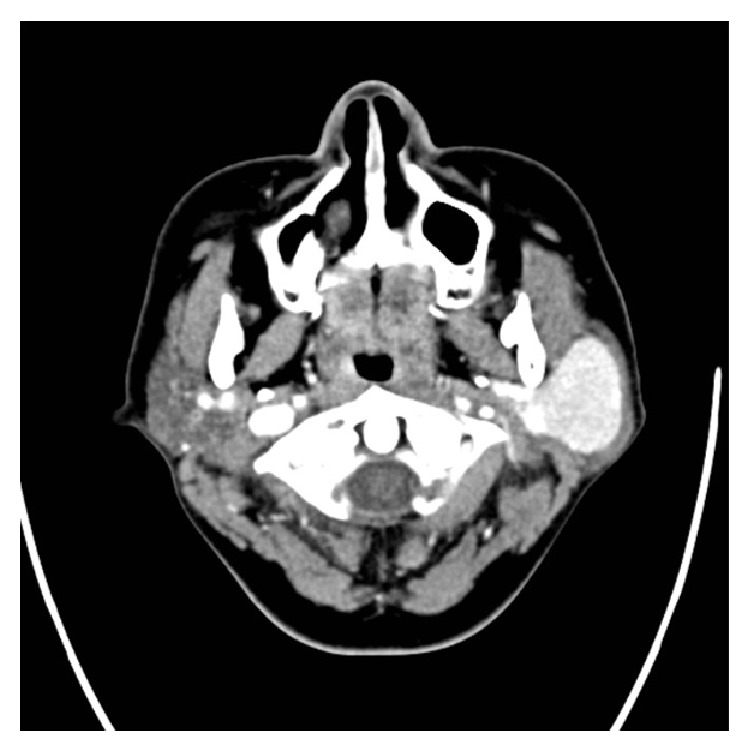
CT head and neck axial cut, after contrast, soft tissue window: a well-defined, oval shaped solid lesion involving the left superficial lobe of the parotid gland. The lesion shows a diffuse intense enhancement after IV contrast media injection.

**Figure 2:**
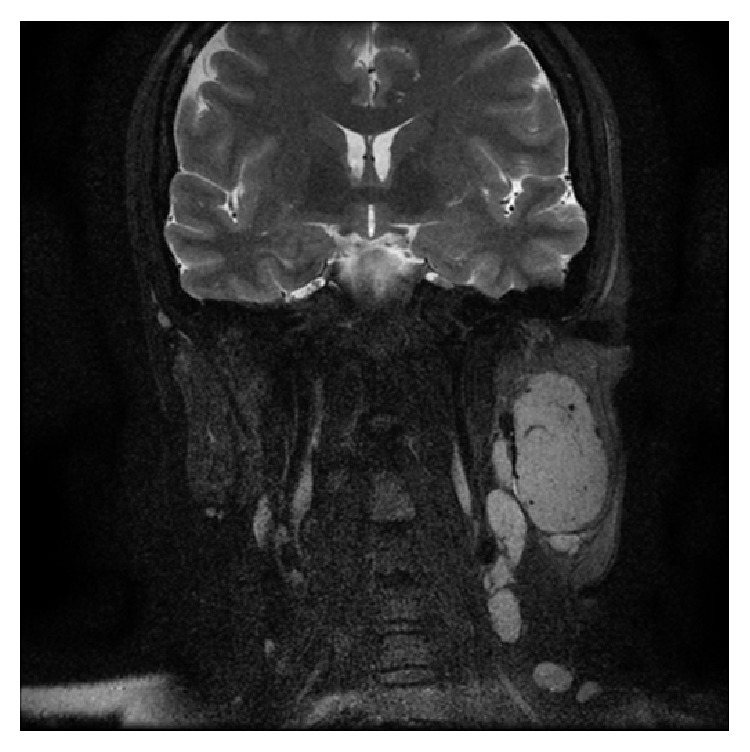
MRI head and neck coronal cut, after contrast, T2: a well-defined, T2 intermediate SI solid lesion in the left superficial parotid lobe. There is diffuse intense enhancement. Multiple enlarged lymph nodes in the left periparotid region.

Preoperatively, the patient underwent a contrasted Computer Tomography (CT) scan of the head and neck that showed a single, well-defined, solid lesion in the superficial lobe of the parotid gland measuring 4.9 × 2.8 × 3.4 cm with a diffuse intense enhancement of the lesion following contrast administration. No calcification or necrotic areas were seen within the lesion. Multiple left periparotid lymph nodes were noted, the largest of which was posterior to the lesion and measured 0.9 × 0.4 cm. All other major salivary glands were unremarkable (Figure 1). The patient further underwent a Magnetic Resonance Imaging (MRI) of the neck that revealed a well-defined, intensely enhancing T1 and T2 intermediate SI and diffusion restriction mass lesion occupying the superficial lobe of the parotid gland with enlarged regional lymph nodes (Figure 2).

Fine-needle aspiration cytology was performed, and the smears showed small activated lymphocytes, few plasma cells, small lymphohistiocytic fragments, and few follicular dendritic cells and eosinophils. Supplementary repeated aspiration for flow cytometry immune-phenotyping was advised. It demonstrated 68% of events in the lymphoid region. Gating after immunostaining showed 98% CD45 expression, with equal T and B lymphocytes and no light chain restriction.

**Figure 3:**
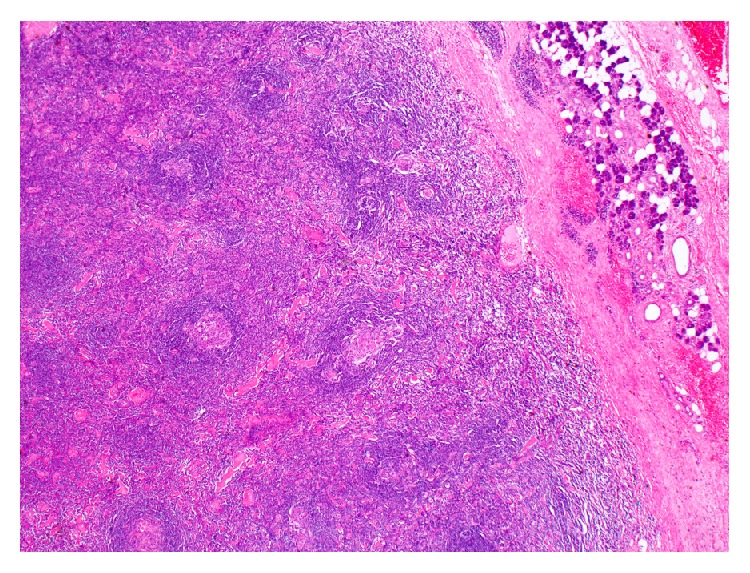
Low magnification of the lesion next to the parotid parenchyma (2x; H&E).

**Figure 4:**
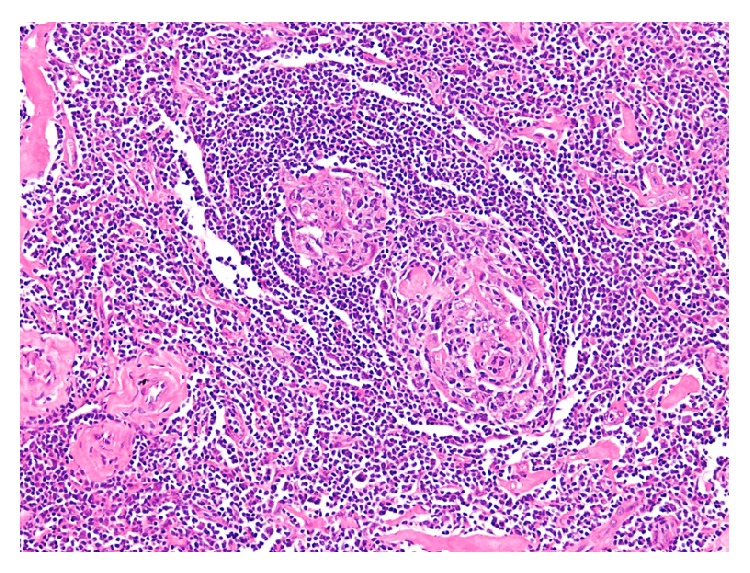
Two burnt-out germinal centers with vascular proliferation within one follicle (10x; H&E).

**Figure 5 fig5:**
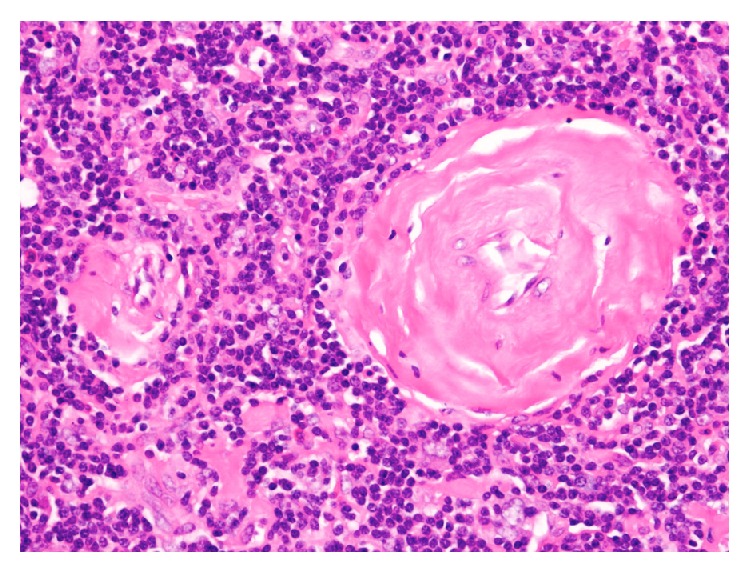
Higher magnification photomicrograph showing the prominent hyalinization around the vessels (20x; H&E).

**Figure 6:**
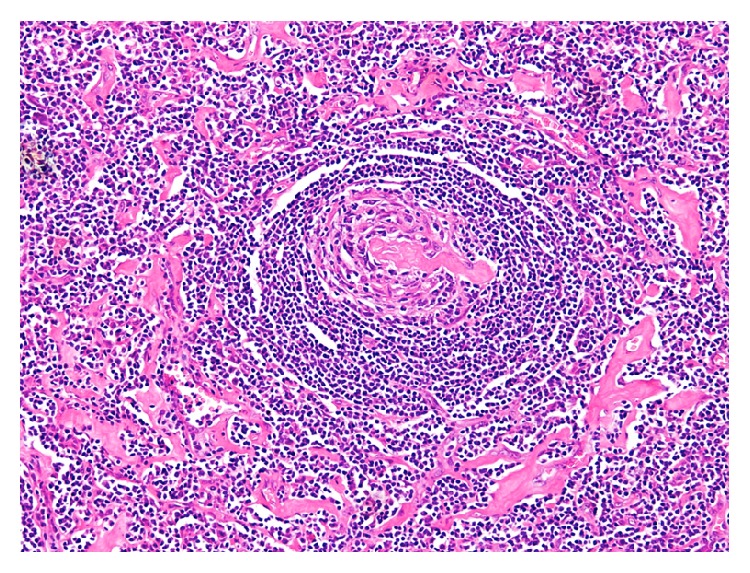
Prominent hyalinization around a vessel entering the germinal center with “onion-skinning” of the mantle zone (10x; H&E).

The patient underwent a left superficial parotidectomy with an intraoperative facial nerve monitoring. Postoperatively the patient was complaining of grade 4 facial palsy (according to House Brackmann grading system). She was treated with dexamethasone and was followed up in our outpatient department. Her facial palsy resolved within a month postoperatively and she had no further complications. Furthermore, she was referred to an oncologist in her home country where she is on regular follow-ups, and she has been disease-free for a period of 12 months. The excised tissue was examined by our head and neck pathologist. Hematoxylin and eosin (H&E) stained sections of the formalin-fixed and paraffin-embedded tissue showed follicular lymphoid hyperplasia though with a rather peculiar morphology (Figure 3). The follicles exhibited vascular proliferation, with prominent hyalinization around the vessels, within burnt-out germinal centers (Figures 4 [Fig fig5]–6). The mantle zone was expanded with the cells somewhat aligned concentrically (Figure 6). One might whimsically say that, with little to no imagination, the overall appearance of the follicle may resemble a lollipop. The diagnosis rendered was angiofollicular lymphoid hyperplasia (Castleman disease), hyaline vascular type.

Postoperatively, a follow-up CT scan of the head and neck showed an unremarkable remaining tissue of the left parotid gland with no residual lesions and normally looking right parotid gland and other salivary gland tissues. Few small normally enhancing lymph nodes were noted in the left levels 1 to 6 and right level 1 B. CT scan of the thoracic-abdominal-pelvic region was unremarkable.

## 3. Discussion

CD was first described by Castleman et al. in 1956, as a benign, localized, enlarged hyperplastic lymph node [1]. This disease has no known etiology, though several theories have been proposed. CD has been classified histopathologically into three subtypes: hyaline vascular, plasma cell, and mixed types. The hyaline vascular CD is the most common type, accounting for 80%–90% of cases [4].

CD is also classified clinically into unicentric or localized and multicentric or generalized types. The multicentric type of CD is more aggressive and it has a predisposition to men in their third to fifth decades of life. The unicentric (localized) form, as the name suggests, has a more benign process [5]. It is usually asymptomatic with just a palpable enlarged lymph node. On the other hand, patients with multicentric (generalized) CD complain of systemic symptoms, including fever, loss of weight, and splenomegaly, and it is usually associated with syndromes such as nephrotic syndrome and POEM's syndrome [6]. Laboratory investigations can help in categorizing CD into the benign and aggressive forms.

Although this disease is still not well understood, several theories have been suggested. Of those, the most supported theory is excessive lymphoproliferation due to chronic stimulation by a virus or chronic inflammation. It has been proven in the literature that there is a strong association between CD and viral infections: EBV, HIV, and HHV-8 [7]. Another strong theory proposes the significance of the interaction between interleukin 6 and tumor necrosis factor alpha and the systemic presentation of multicentric CD [7].

Generally, the management of CD depends on the type. The benign localized form is usually treated with local excision of the lesion [8]. However the nonoperable cases are managed with radiotherapy although excision has a more preferable prognosis. On the contrary and due to the aggressiveness of the multicentric form, it is usually controlled by palliative treatment only [7]. Some patients require corticosteroid therapy with occasional chemotherapy in nonresponders to steroid [7]. The most important step in the management is the long follow-up period due to the possibility of malignant transformation.

## 4. Conclusion

CD is a rare lymphoproliferative disorder that has no specific clinical, radiological, or cytological features. It is diagnosed by exclusion with the aid of histopathological examination. Although extremely rare in the head and neck, CD should always be a part of the differential diagnosis list of any head and neck swelling, especially when the FNAC findings coupled with the clinical presentation hint at it.
